# Vulnerability to HIV Infection Among International Immigrants in China: Cross-sectional Web-Based Survey

**DOI:** 10.2196/35713

**Published:** 2023-01-10

**Authors:** Yuyin Zhou, Feng Cheng, Junfang Xu

**Affiliations:** 1 Center for Health Policy Studies School of Public Health Zhejiang University School of Medicine Hangzhou China; 2 Department of Pharmacy Second Affiliated Hospital Zhejiang University School of Medicine Hangzhou China; 3 Vanke School of Public Health Tsinghua University Beijing China; 4 Institute for Healthy China Tsinghua University Beijing China

**Keywords:** international immigrants, HIV, risky sexual behavior, China

## Abstract

**Background:**

The rising number of migrants worldwide, including in China given its recent rapid economic development, poses a challenge for the public health system to prevent infectious diseases, including sexually transmitted infections (STIs) caused by risky sexual behaviors.

**Objective:**

The aim of this study was to explore the risky sexual behaviors of international immigrants living in China to provide evidence for establishment of a localized public health service system.

**Methods:**

Risky sexual behaviors were divided into multiple sexual partners and unprotected sexual behaviors. Basic characteristics, sexual knowledge, and behaviors of international immigrants were summarized with descriptive statistics. Multivariate logistic regression analyses were used to identify factors associated with risky sexual behaviors, and the associations of demographic characteristics and risk behaviors with HIV testing and intention to test for HIV.

**Results:**

In total, 1433 international immigrants were included in the study, 61.76% (n=885) of whom had never heard of STIs, and the mean HIV knowledge score was 5.42 (SD 2.138). Overall, 8.23% (118/1433) of the participants had been diagnosed with an STI. Among the 1433 international immigrants, 292 indicated that they never use a condom for homosexual sex, followed by sex with a stable partner (n=252), commercial sex (n=236), group sex (n=175), and casual sex (n=137). In addition, 119 of the international immigrants had more than three sex partners. Individuals aged 31-40 years were more likely to have multiple sexual partners (adjusted odds ratio [AOR] 2.364, 95% CI 1.149-4.862). Married participants were more likely to have unprotected sexual behaviors (AOR 3.096, 95% CI –1.705 to 5.620), whereas Asians were less likely to have multiple sexual partners (AOR 0.446, 95% CI 0.328-0.607) and unprotected sexual behaviors (AOR 0.328, 95% CI 0.219-0.492). Women were more likely to have taken an HIV test than men (AOR 1.413, 95% CI 1.085-1.841). Those who were married (AOR 0.577, 95% CI 0.372-0.894), with an annual disposable income >150,000 yuan (~US $22,000; AOR 0.661, 95% CI 0.439-0.995), considered it impossible to become infected with HIV (AOR 0.564, 95% CI 0.327-0.972), and of Asian ethnicity (AOR 0.330, 95% CI 0.261-0.417) were less likely to have an HIV test. People who had multiple sexual partners were more likely to have taken an HIV test (AOR 2.041, 95% CI 1.442-2.890) and had greater intention to test for HIV (AOR 1.651, 95% CI 1.208-2.258).

**Conclusions:**

International immigrants in China exhibit risky sexual behaviors, especially those aged over 30 years. In addition, the level of HIV-related knowledge is generally low. Therefore, health interventions such as targeted, tailored programming including education and testing are urgently needed to prevent new HIV infections and transmission among international immigrants and the local population.

## Introduction

Under globalization, the number of international travelers has increased significantly worldwide during the past decade, from 2433 million in 2008 to 3188 million in 2019 [1,2]. Owing to its rapid economic development, China has become a popular country and attracted numerous international immigrants. For example, the National Bureau of Statistics showed that approximately 845,697 international immigrants lived in China in 2020, increasing by 251,865 compared to that recorded in 2010 [3,4]. Thus, an increasing number of international immigrants settled in China with different ethnic backgrounds and cultures, and this significant number of immigrants can inevitably cause some public health problems [5]. The health challenges posed by migration, not only for the immigrants themselves but also for residents, have become an important global health issue. For example, migration has proven to be a risk factor for sexually transmitted infections (STIs), especially with respect to the spread of HIV to residents of the destination country during unprotected sexual encounters [6]. Risky sexual behaviors might increase upon immigration given the removal of factors that might inhibit sexual freedom in the home country, including physical separation of partners or social networks, escaping from the pressure of home community or social culture, and especially an increase in casual sex [7].

The total number of people living with HIV/AIDS worldwide increased from 31.1 million in 2010 to 37.7 million in 2020 [8]. However, new HIV infections have declined by 31%, from 2.1 million in 2010 to 1.5 million in 2020 [8]. Migration has become one of the key issues related to HIV prevention and control in recent years considering the increasing number of HIV infections among migrants [9]. Indeed, since the beginning of the HIV epidemic, governments have been worried that migrants may be largely responsible for spreading HIV [10,11]. However, specific public health responses have not yet been established to monitor HIV among migrants, especially international immigrants, in most countries. Considering that the majority of international immigrants are sexually active young people and their interaction with local Chinese residents is inevitable, active responses for public health and related research are particularly important.

In China, the number of newly reported HIV-infected international immigrants reached up to 15,319 between 2004 and 2017 [12], although there is currently no mandatory screening for HIV or other STIs for international immigrants in China. Most studies regarding the sexual behaviors of international immigrants have been conducted in high-income countries such as the United States, Canada, and the United Kingdom [13-15]. The status of sexual behaviors among international immigrants living in China is currently unclear. Moreover, considering the differences in cultures and health services, interventions that are employed in high-income countries may not be suitable for international immigrants in China. Therefore, we aimed to explore the risky sexual behaviors of international immigrants living in China to provide evidence for establishment of a localized public health service system to address the significant increase in the number of international immigrants under globalization.

## Methods

### Participants

We used a cross-sectional web-based study design to collect data from international immigrants living in China by a snowball sampling method. Three to six international immigrants with different occupations, ages, home countries, and genders from Yiwu, Guangzhou, Beijing, and Hangzhou were invited to start the investigation. These immigrants were then asked to invite other international immigrants living in China to participate in the survey to expand the sample. We selected these locations in particular because Yiwu, as one of the largest commodity trading cities, attracts nearly half a million international immigrants every year [16]; Guangzhou is located in the core of the Pearl River Delta [17], and is also an important trading port representing one of the cities receiving a significant number of international immigrants; Beijing is the capital of China, which is bound to attract a substantial number of foreign people as the political center; and Hangzhou is the provincial capital of Zhejiang and located in the eastern coast of China, which has developed rapidly in recent years because of the high-tech service industry, resulting in a large foreign population.

The inclusion criteria of participants were: (1) international immigrants whose homeland was not China but were living in China, (2) above 18 years old, (3) could read English, and (4) willing to participate in the study. The exclusion criteria were: (1) under 18 years old; (2) Chinese nationality; and 3) did not pass the “attention check” in the questionnaire, which was used to identify careless respondents and improve the data quality.

The sample size was calculated based on the 31.14% HIV diagnosis risk among international immigrants according to previous studies [11,18], which required at least 330 participants. We also estimated the sample size based on 5-10 times the number of questions in the questionnaire, which required 650 respondents. To select the maximum representative sample from the population, 1460 participants were recruited and the responses of 1433 international immigrants were incorporated in the final analysis.

### Data Collection

Considering the sensitivity of sexual issues and the COVID-19 pandemic, we used an online questionnaire rather than a face-to-face survey, which was conducted between January and September 2021, including sociodemographic information (eg, gender, age, marital status, education level, employment, and annual disposable income), HIV-related knowledge, STIs history (eg, diagnosis of genital herpes, syphilis, and condyloma in the past), HIV testing history (ie, have taken an HIV test in the past), and intention to have an HIV test in the future.

In addition, all participants were invited to recall their related sexual behaviors while living in China in the past year. Sexual behavior–related data included the number of sexual partners; condom use; stable, casual, commercial, homosexual, and group sexual behaviors; as well as illicit drug use. Risky sexual behaviors were classified as having multiple sexual partners and unprotected sexual behaviors. HIV-related knowledge was measured using the 8-item HIV Knowledge Questionnaire (HIV-KQ-8) [19]. If the answer was correct, a score of 1 was assigned, whereas a score of 0 was given if the answer was wrong or “do not know.” HIV/STIs knowledge was finally evaluated by the total scores, with a maximum score of 8 if all answers were correct; thus, a higher score represented a higher level of HIV/STIs knowledge among international immigrants. The questions regarding sexual behaviors were designed based on the guidelines of intervention work for the prevention of HIV/AIDS issued by the China Center for Disease Control and our previous study [20,21]. The reliability and validity of the questionnaires were confirmed using Cronbach α (.996) and the Kaiser-Meyer-Olkin test (0.961).

### Ethics Considerations

The study protocol and consent procedure were approved by the Ethics Review Committee, School of Public Health, Zhejiang University (#2019-064). Informed consent information was provided before the questions; participants had the option to exit the survey after reading the informed consent information or to provide consent to continue. The confidentiality of individuals was properly protected in the management of the investigation and the processing of data.

### Statistical Analysis

Descriptive statistics were used to analyze the basic characteristics, sexual-related knowledge, and sexual behaviors of international immigrants using frequency and percentage or mean (SD) as appropriate. Univariate and multivariate logistic regression analyses were used to identify the factors associated with risky sexual behaviors among international immigrants and the association with HIV test history and intention to receive an HIV test in the future. SPSS 24.0 statistical software (IBM, Armonk, NY, USA) was used to analyze all data. *P*<.05 was considered to indicate a statistically significant association.

## Results

### Basic Characteristics of International Immigrants in China

In total, 1433 international immigrants were included in the study, with a predominance of men (Table 1). The average age was approximately 25 years and most of the participants were well-educated with 68.95% (988/1433) reporting more than 11 years of education. The annual disposable income of the majority of the international immigrants was less than 50,000 yuan (~US $7200). The majority of the respondents were unmarried, and the largest proportion of immigrants were from Africa, followed by America, Europe, and Asia (Table 1).

**Table 1:** Basic characteristics of international immigrants in China (N=1433).

Characteristics		Value
**Gender, n (%)**		
	Male	973 (67.90)
	Female	460 (32.10)
Age (years), mean (SD)		24.97 (4.57)
**Marital status, n (%)**		
	Unmarried	1263 (88.14)
	Married	110 (7.68)
	Windowed	4 (0.28)
	Divorced	9 (0.63)
	Other	47 (3.28)
**Education level, n (%)**		
	Illiterate	49 (3.42)
	1-5 years	299 (20.87)
	6-10 years	97 (6.77)
	11-12 years	157 (10.96)
	>12 years	831 (58.00)
**Employment, n (%)**		
	Employed	231 (16.12)
	Unemployed	1202 (83.88)
**Annual disposable income (yuan)^a^, n (%)**		
	≤50,000	1040 (72.58)
	50,001-100,000	212 (14.79)
	100,001-150,000	79 (5.51)
	>150,000	102 (7.12)
**Home continent^b^, n (%)**		
	Africa	679 (47.38)
	America	67 (4.68)
	Europe	40 (2.79)
	Asia	610 (42.57)
	Other	6 (0.42)

^a^Exchange rate on December 7, 2021: 6.3708 yuan=US $1.

^b^There were 31 responses missing.

### Knowledge on HIV Among International Immigrants in China

Among the 1433 international immigrants, 61.76% (885) had never heard of STIs or HIV. Among the immigrants who had heard of HIV/STIs, the mean knowledge score on HIV was 5.42 (SD 2.138). The percentage of respondents with an HIV knowledge score <4, 4-6, and >6 was 6.98% (100/1433), 17.31% (248/1433), and 13.89% (199/1433), respectively.

### Self-Report of STIs Among International Immigrants

Overall, 91.77% (1315/1433) of international immigrants had not been diagnosed with any STIs, whereas 8.23% (118/1433) of the international immigrants had been diagnosed with at least one STI, including gonorrhea (16/1433, 1.12%), syphilis (12/1433, 0.84%), condyloma acuminatum (1/1433, 0.07%), herpes progenitalis (3/1433, 0.21%), hepatitis B (9/1433, 0.63%), and hepatitis C (2/1433, 0.14%). In addition, 4.82% (69/1433) of the participants had been diagnosed with HIV.

### Risky Sexual Behaviors

**Figure 1:**
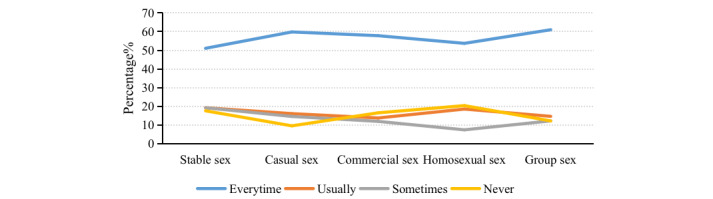
Frequency of condom use according to sexual behavior among international immigrants.

As shown in Figure 1, more than 50% of international immigrants reported using a condom during sexual behaviors. However, 20.38% (292/1433) of international immigrants engaging in homosexual sex reported never using condoms, followed by 17.59% (252/1433) in stable sexual relationships, 16.47% (236/1433) engaging in commercial sex, 12.21% (175/1433) for group sex, and 9.56% (137/1433) for casual sex. In addition, 8.30% (119/1433) of international immigrants had more than three sexual partners.

### Factors Associated With Risky Sexual Behaviors Among International Immigrants

The binary logistic regression model showed the factors affecting whether the respondents had multiple partners or unprotected sex as a risky sexual behavior (see Table S1 in Multimedia Appendix 1). We found that being aged 31-40 years was significantly associated with having multiple partners (adjusted odds ratio [AOR] 2.364, 95% CI 1.149-4.862). Married respondents indicated more unprotected risk behaviors (AOR 3.096, 95% CI –1.705 to 5.620) compared with unmarried respondents. Asians tended to have fewer multiple partners (AOR 0.446, 95% CI 0.328-0.607) and unprotected risk behaviors (AOR 0.328, 95% CI 0.219-0.492) compared with Africans.

### Demographic Characteristics, Risk Behaviors, and Their Association With HIV Testing and Intention to Test for HIV

Table S2 in Multimedia Appendix 1 shows the associations of demographic characteristics and risk behaviors with HIV testing. Women had a higher probability of having taken an HIV test in the past than men (AOR 1.413, 95% CI 1.085-1.841). People who were married had less intention to take an HIV test (AOR 0.577, 95% CI 0.372-0.894) compared to unmarried respondents. Those considering it to be impossible to become infected with HIV were less likely to have ever taken an HIV test (AOR 0.523, 95% CI 0.281-0.975) or to intend to take an HIV test (AOR 0.564, 95% CI 0.327-0.972) compared with those who thought it could be possible to become infected. Compared to respondents who did not have multiple sex partners, those who reported having multiple sexual partners were more likely to intend to test for HIV (AOR 1.797, 95% CI 1.324-2.438). Furthermore, Asians were less likely to indicate an intention to be tested for HIV (AOR 0.330, 95% CI 0.261-0.417). 

## Discussion

### Principal Findings

International immigrants in China exhibited risky sexual behaviors, with 22.40% and 13.19% of the respondents reporting having multiple sexual partners and unprotected sexual behaviors, respectively. These trends may be partly related to the differences in the new living environment faced by international immigrants from those of the homeland. For example, the absence of family and peer monitoring, language barriers, cultural barriers, and a sense of anonymity may offer immigrants more sexual freedom [22,23]. In addition, 4.82% of the respondents reported that they have been diagnosed with HIV. Therefore, the risk of HIV transmission and infection among international immigrants exists, and interventions are urgently needed to control the HIV epidemic caused by international migration in China.

HIV knowledge is a significant factor contributing to HIV prevention [14]. Previous studies also showed that enhanced knowledge moderated sexual risk behaviors, and HIV knowledge increased safer-sex intentions, condom use, abstinence, and HIV testing and treatment [24-27]. However, the majority of the international immigrants surveyed in our study had never heard of STIs, and the knowledge scores of the participants who were aware of HIV were also quite low. A study in Canada suggested that those born in sub-Saharan African also scored relatively low on the HIV knowledge questionnaire, which was consistent with our results [14]. Based on our analysis between Chinese nationals and international immigrants, the mean score of HIV-related knowledge of international immigrants who had heard of HIV (5.42, SD 2.14) was lower than that of Chinese nationals (6.68, SD 2.08), and there was a much higher number of international immigrants who had never heard of HIV (61.76%) compared to Chinese nationals (5.48%). These results were consistent with previous research [28]. The reasons may include that immigrants represent a marginalized group, who may not receive any HIV education in either their homeland or destination country. However, there are also discrepancies among studies related to the extent of HIV knowledge among immigrants. For example, a study in Thailand indicated that immigrants exhibited a fairly high standard of knowledge about the risk factors of HIV [5], and this knowledge increased among immigrants who had heard of HIV after they came to Thailand, which also showed that they obtained adequate education from the local government. Another study performed in South Korea also found that immigrant workers exhibited better knowledge of HIV compared with Korean respondents [29]. These differences may be caused by the fact that the participants in our study mostly migrated from low- and middle-income countries, where HIV-related resources are limited.

The age group of 30-60 years was significantly associated with having more risky sexual behaviors among the international immigrants surveyed in our study, which was consistent with the majority of previous related studies. This may be related to the fact that this age corresponds to a more physiologically sexually active group, who may have sex with multiple partners without protection. A previous study published in 2018 also reached a similar conclusion, showing that adults of this age group were more likely to have sex without a condom, new sexual partners, and multiple sexual partners [30]. In addition, consistent with a prior study showing that married people tended to have more high-risk sexual behaviors [31], we found that married individuals tended to have more unprotected sexual behaviors than unmarried individuals. There are several possible reasons for this difference. For example, a traditional culture that does not permit engaging in sexual behaviors before marriage could cause more risky sexual behaviors among married than unmarried individuals. In addition, married people may plan to have a baby, which would be accompanied by unprotected sex [32]. Moreover, some people find it shameful to use condoms once married or are pursuing the physical and psychological satisfaction from unprotected sex [13]. However, in our study, 16.13% of the married respondents indicated engaging in unprotected sexual behaviors not with their stable partners. For instance, a prior study also showed that married immigrant men tended to have affairs [33], and 28.48% of the migrant workers who lived apart from their spouses had extramarital sexual behaviors [34]. People engaging in casual sex, commercial sex, homosexual sex, and group sex are considered to be a high-risk population for STIs, and the frequency of condom use was rather low among international immigrants; therefore, health education is necessary and important for these international immigrants. Moreover, the environment of field testing for HIV typically has poor privacy protection, which may lead those at high risk of worrying about disclosure of personal information, especially for international immigrants who may be more insecure about whether they will be able to settle in the destination country if they are diagnosed with HIV.

With respect to the influence of the origin region of the immigrants, we found that Asians exhibited less risky sexual behaviors but also had reduced intention to be tested for HIV compared with Africans. The possible reasons for these differences may include that polygamy still exists in some areas of Africa, especially in some sub-Saharan African countries [35], and international immigrants from Africa tend to have little knowledge about HIV [36,37]. These factors may influence their sexual behaviors, especially increasing the risk of having multiple sexual partners and unprotected sexual behaviors. In addition, the fact that Africa had the highest number of people living with HIV in 2020, suggesting that Africans are most heavily affected by HIV [38], may cause their higher intention to receive an HIV test in the future compared with Asians. Among international immigrants, homosexuals were less likely to have ever tested for HIV than heterosexuals. Generally, homosexuals are considered to engage in risky sexual behaviors. Without an HIV test, the infection status would be unknown, which would place their partners at greater risk for the transmission of HIV and other STIs. Moreover, the proportion of HIV infections transmitted through homosexual behaviors has increased significantly in recent years [31]; thus, there is an urgent need to promote HIV testing among this high-risk group.

### Limitations

This study had some limitations. First, it is possible that the participants were influenced by social and cultural norms to hide their risky sexual behavior, including a history of commercial or casual sexual behaviors, which may affect the research results, although an online survey could avoid the embarrassment caused by a face-to-face survey. Second, although the seed participants were selected to have different occupations, ages, homelands, and genders from different cities, a bias caused by snowball sampling may still exist, which could lead to misinterpretation of the related statistical results. In addition, considering the significant number of international immigrants living in China, our participants were mainly from lower socioeconomic strata with a lower annual disposable income, which represents a narrow band of the socioeconomic system. Therefore, the results may not be generalizable to the whole population. Third, the questionnaire regarding risky sexual behaviors was designed based on previous work and was amended according to our research participants and objectives. Thus, the validity of the questionnaire in the survey should be further tested.

### Implications for Health Policy

Globalization leads to an inevitable trend of increased population mobility. Under this situation, it is necessary to take proactive public health approaches to manage the health of international immigrants, especially with respect to infectious diseases. Our study showed that the overall HIV-related knowledge of the international immigrants in China was low along with a low rate of intention to test for HIV. Therefore, HIV-related education is essential, such as by community advocacy and online propaganda delivered in their native languages, to address the gaps in HIV knowledge among international immigrants. More importantly, it is difficult for international immigrants to access HIV-related services because of the lack of specified health care and prevention upon arrival in the new country, which may cause immigrants with an HIV-positive status to remain undiscovered until some typical symptoms appear. To prevent the transmission of HIV, the basic public health care services provided by the Chinese government should be extended to cover international immigrants. Active promotion for antiviral treatment is less available for international immigrants with HIV infection than for local residents in China. Thus, an integrated health system for HIV prevention, monitoring, and treatment for immigrants and local residents will be important to reduce the transmission rate of HIV. Moreover, this is also necessary to reduce the inequities in access to health care and health outcomes for immigrants. It is also important to consider the wider health care and socioeconomic barriers immigrants face to be successful in improving HIV outcomes. In addition, as a convenient and efficient method, HIV self-testing could be promoted among international immigrants, such as setting up rapid detection kit vending machine in immigrant gathering communities [39]. Moreover, online measures, including HIV testing, education, and interventions, could be developed using the internet and big data to solve the privacy issues that are a major concern of international immigrants. 

### Conclusion

Risky sexual behaviors exist among international immigrants in China, including multiple partners and unprotected sexual behaviors, and the level of cognition of HIV knowledge was quite low, especially for married immigrants and those aged above 30 years. Therefore, there is an urgent need to promote health education and HIV testing specified for international immigrants. In addition, it is also important to develop and improve health care access for international immigrants under the current situation of increasing globalization.

## Appendix

Multimedia Appendix 1 Factors associated with risky sexual behaviors (Table S1), and demographic characteristics, risk behaviors, and their association with HIV testing and intention to test for HIV (Table S2) among international immigrants.

## Abbreviations

AOR: adjusted odds ratio
HIV-KQ-8: 8-item HIV Knowledge Questionnaire
STI: sexually transmitted infection

## Availability of Data and Material

The views expressed in this publication do not represent the views of the Veterans Health Administration or the United States Government.
