# Supplementary material for: Vulnerability to HIV Infection Among International Immigrants in China: Cross-sectional Web-Based Survey
Source: JMIR Public Health Surveill. 2023 Jan 10;9:e35713. doi: 10.2196/35713 (PMC9874985; doi:10.2196/35713)
Supplement: Multimedia Appendix 1 [file publichealth_v9i1e35713_app1.docx]

Table S1. Factors associated with risky sexual behaviors among international immigrants (N=1433).

| Variables | | Having multiple sexual partners (No=0, Yes=1) | | | | | Having unprotected risk behaviors (No=0, Yes=1) | | | | |
| --- | --- | --- | --- | --- | --- | --- | --- | --- | --- | --- | --- |
|  | | Responses, n (%) | Crude OR (95% CI) | *P* value | Adjusted OR (95% CI) | *P* value | Response, n (%) | Crude OR (95% CI) | *P* value | Adjusted OR (95% CI) | *P* value |
| **Gender** | | | | | | | | | | | |
|  | Male | 224 (23.0) | reference |  |  |  | 120 (12.3) | reference |  |  |  |
|  | Female | 97 (21.1) | 0.894 (0.683-1.170) | .41 | 0.825 (0.617-1.103) | .19 | 69 (15.0) | 1.254 (0.911-1.726) | .16 | 1.270 (0.898-1.797) | .18 |
| Age group (years) | | | | | | | | | | | |
|  | ≤20 | 11 (12.2) | Reference |  |  |  | 7 (7.8) | reference |  |  |  |
|  | 21-30 | 178 (19.7) | 1.763 (0.919-3.383) | .09 | 1.644 (0.825-3.277) | .16 | 100 (11.1) | 1.477 (0.664-3.283) | .34 | 1.421 (0.608-3.325) | .42 |
|  | 31-40 | 86 (28.1) | 2.807 (1.425-5.532) | *.003* | 2.364 (1.149-4.862) | *.02* | 54 (17.6) | 2.541 (1.113-5.801) | *.03* | 2.060 (0.851-4.985) | .11 |
|  | 40-60 | 46 (34.3) | 3.754 (1.819-7.748) | *<.001* | 2.460 (1.086-5.571) | *.03* | 28 (20.9) | 3.132 (1.304-7.526) | *.01* | 1.516 (0.551-4.165) | .42 |
| **Marital status** | | | | | | | | | | | |
|  | Unmarried | 273 (21.6) | reference |  |  |  | 147 (11.6) | reference |  |  |  |
|  | Married | 35 (31.8) | 1.692 (1.109-2.583) | *.02* | 1.385 (0.813-2.358) | .23 | 31 (28.2) | 2.979 (1.900-4.670) | *<.001* | 3.096 (–1.705-5.620) | *<.001* |
|  | Windowed | 2 (50) | 3.626 (0.508-25.862) | .20 | 2.052 (0.175-24.018) | .57 | 2 (50.0) | 7.592 (1.061-54.302) | *.04* | 3.880 (0.314-47.900) | .29 |
|  | Divorced | 2 (22.2) | 1.036 (0.214-5.016) | .97 | 0.648 (0.117-3.580) | .62 | 2 (22.2) | 2.169 (0.446-10.540) | .34 | 1.711 (0.310-9.463) | .54 |
|  | Other | 9 (19.1) | 0.859 (0.410-1.798) | .69 | 0.911 (0.420-1.976) | .81 | 7 (14.9) | 1.329 (0.584-3.020) | .50 | 1.202 (0.509-2.839) | .68 |
| **Education level** | | | | | | | | | | | |
|  | Illiteracy | 11 (22.4) | Reference |  |  |  | 8 (16.3) | reference |  |  |  |
|  | 1-5 years | 76 (25.4) | 1.177 (0.573-2.418) | .66 | 1.607 (0.731-3.533) | .24 | 44( 14.7) | 0.884 (0.389-2.013) | .77 | 1.117 (0.454-2.751) | .81 |
|  | 6-10 years | 12 (12.4) | 0.488 (0.198-1.203) | .12 | 0.500 (0.188-1.327) | .16 | 10 (10.3) | 0.589 (0.216-1.603) | .30 | 0.567 (0.190-1.691) | .31 |
|  | 11-12years | 29 (18.5) | 0.783 (0.358-1.712) | .54 | 1.086 (0.460-2.565) | .85 | 15 (9.6) | 0.541 (0.215-1.366) | .19 | 0.739 (0.268-2.036) | .56 |
|  | ＞12years | 193 (23.2) | 1.045 (0.524-2.084) | .90 | 1.326 (0.616-2.858) | .47 | 112 (13.5) | 0.798 (0.365-1.747) | .57 | 1.073 (0.448-2.569) | .88 |
| **Employment** | | | | | | | | | | | |
|  | Employed | 79 (34.2) | reference |  |  |  | 47 (20.3) | reference |  |  |  |
|  | Unemployed | 242 (20.1) | 0.485 (0.357-0.659) | *<.001* | 0.570 (0.393-0.827) | *.003* | 142 (11.8) | 0.524 (0.364-0.756) | *.001* | 0.728 (0.464-1.143) | .17 |
| **Annual disposable income** | | | | | | | | | | | |
|  | ≤50000 | 225 (21.6) | reference |  |  |  | 127 (12.2) | reference |  |  |  |
|  | 50001-100000 | 55 (25.9) | 1.269 (0.903-1.784) | .17 | 1.188 (0.819-1.722) | .37 | 37 (17.5) | 1.520 (1.019-2.268) | *.04* | 1.460 (0.939-2.268) | .09 |
|  | 100001-150000 | 17(21.5) | 0.993 (0.569-1.733) | .98 | 0.784 (0.431-1.426) | .43 | 14 (17.7) | 1.548 (0.844-2.840) | .16 | 1.218 (0.628-2.363) | .56 |
|  | ＞150000 | 24 (23.5) | 1.115 (0.689-1.802) | .66 | 0.954 (0.562-1.620) | .86 | 11 (10.8) | 0.869 (0.452-1.669) | .67 | 0.658 (0.323-1.339) | .25 |
| **Perceived risk of being infected with HIV** | | | | | | | | | | | |
|  | Sure | 22 (37.3) | reference |  |  |  | 15 (25.4) | reference |  |  |  |
|  | Very likely | 16 (43.2) | 1.281 (0.555-2.961) | .56 | 1.555 (0.629-3.849) | .34 | 12 (32.4) | 1.408 (0.570-3.477) | .46 | 2.069 (0.768-5.576) | .15 |
|  | Possible | 20 (21.3) | 0.455 (0.221-0.936) | *.03* | 0.468 (0.217-1.011) | .05 | 16 (17.0) | 0.602 (0.272-1.333) | .21 | 0.706 (0.298-1.674) | .43 |
|  | Unlikely | 107 (26.8) | 0.614 (0.347-1.088) | .10 | 0.575 (0.304-1.085) | .09 | 61 (15.3) | 0.528 (0.277-1.007) | *.05* | 0.538 (0.259-1.117) | .10 |
|  | Impossible | 156 (18.5) | 0.382 (0.219-0.666) | *.001* | 0.395 (0.214-0.728) | *.003* | 85 (10.1) | 0.329 (0.176-0.616) | *.001* | 0.397 (0.197-0.802) | *.01* |
| **Sexual orientation** | | | | | | | | | | | |
|  | Homosexual | 48 (21.1) | 0.858 (0.605,1.217) | .39 | 0.947 (0.637-1.410) | .79 | 30 (13.2) | 0.976 (0.638-1.492) | .91 | 1.086 (0.662-1.783) | .74 |
|  | Bisexual | 33 (17.1) | 0.663 (0.444,0.991) | *.05* | 0.717 (0.464-1.108) | .13 | 23 (11.9) | 0.871 (0.544-1.396) | .57 | 0.964 (0.577-1.611) | .89 |
|  | Heterosexual | 240 (23.7) | reference |  |  |  | 136 (13.4) | reference |  |  |  |
| **Home continent** | | | | | | | | | | | |
|  | Africa | 186 (27.4) | reference |  |  |  | 115 (16.9) | reference |  |  |  |
|  | America | 22 (32.8) | 1.296 (0.757-2.217) | .34 | 1.024 (0.568-1.845) | .94 | 14 (20.9) | 1.295 (0.695-2.413) | .42 | 0.864 (0.430-1.739) | .68 |
|  | Europe | 13 (32.5) | 1.276 (0.645-2.526) | .48 | 1.402 (0.673-2.919) | .37 | 8 (20.0) | 1.226 (0.551-2.729) | .62 | 1.196 (0.512-2.792) | .68 |
|  | Asia | 96 (15.7) | 0.495 (0.376-0.652) | *<.001* | 0.446 (0.328-0.607) | *<.001* | 48 (7.9) | 0.419 (0.293-0.598) | *<.001* | 0.328 (0.219-0.492) | *<.001* |
|  | Other | 2 (33.3) | 1.325 (0.241-7.296) | .75 | 0.950 (0.154-5.861) | .96 | 2 (33.3) | 2.452 (0.444-13.547) | .30 | 1.355 (0.202-9.069) | .75 |

Table S2. Demographic characteristics, risk behaviors, and their association with HIV testing and intention to test for HIV (N=1433).

| Variables | | Have ever tested for HIV (No=0, Yes=1) | | | | | Will test for HIV in the future (No=1, Doesn’t matter=2, Yes=3) | | | | |
| --- | --- | --- | --- | --- | --- | --- | --- | --- | --- | --- | --- |
|  |  | Yes, n (%) | COR (95% CI) | *P* value | AOR (95% CI) | *P* value | Yes, n (%) | COR (95% CI) | *P* value | AOR (95% CI) | *P* value |
| **Gender** | | | | | | | | | | | |
|  | Male | 261 (26.8) | reference |  |  |  | 358 (36.8) | reference |  |  |  |
|  | Female | 159 (34.6) | 1.441 (1.135-1.830) | *.003* | 1.413 (1.085-1.841) | *.01* | 172 (37.4) |  |  | 1.045 (0.837-1.305) | .70 |
| **Age group (years)** | | | | | | | | | | | |
|  | ≤20 | 11 (12.2) | reference |  |  |  | 27 (30.0) | reference |  |  |  |
|  | 21-30 | 263 (29.1) | 2.951 (1.546-5.636) | *.001* | 2.895 (1.418-5.909) | *.003* | 338 (37.4) |  |  | 1.421 (0.924-2.184) | .11 |
|  | 31-40 | 100 (32.7) | 3.486 (1.776-6.844) | *<.001* | 2.812 (1.334-5.924) | *.007* | 116 (37.9) |  |  | 1.261 (0.788-2.019) | .33 |
|  | 40-60 | 46 (34.3) | 3.754 (1.819-7.748) | *<.001* | 3.122 (1.347-7.232) | *.008* | 49 (36.6) |  |  | 1.404 (0.788-2.502) | .25 |
| **Marital status** | | | | | | | | | | | |
|  | Unmarried | 369 (29.2) | reference |  |  |  | 483 (38.2) | reference |  |  |  |
|  | Married | 29 (26.4) | 0.867 (0.558-1.348) | .53 | 0.781 (0.448-1.361) | .38 | 32 (29.1) | 0.587 (0.408-0.844) | *.004* | 0.577 (0.372-0.894) | *.01* |
|  | Windowed | 1 (25.0) | 0.808(0.084-7.789) | .85 | 1.209 (0.092-15.949) | .89 | 1 (25) | 0.616 (0.099-3.818) | .60 | 0.759 (0.088-6.567) | .80 |
|  | Divorced | 5 (55.6) | 3.028 (0.809-11.341) | .10 | 2.347 (0.512-10.764) | .27 | 3 (33.3) | 0.813 (0.240-2.756) | .74 | 0.886 (0.227-3.461) | .86 |
|  | Other | 16 (34.0) | 1.250 (0.676-2.314) | .48 | 1.133 (0.584-2.198) | .71 | 11 (23.4) | 0.647 (0.376-1.112) | .12 | 0.487 (0.276-0.859) | *.01* |
| **Education level** | | | | | | | | | | | |
|  | Illiterate | 13 (26.5) | reference |  |  |  | 12 (24.5) | reference |  |  |  |
|  | 1-5 years | 91 (30.4) | 1.212 (0.614-2.392) | .58 | 1.262 (0.593-2.686) | .55 | 120 (40.1) |  |  | 2.168 (1.179-3.985) | *.01* |
|  | 6-10 years | 22 (22.7) | 0.812 (0.368-1.794) | .61 | 0.845 (0.352-2.029) | .71 | 34 (35.1) |  |  | 1.872 (0.938-3.735) | .08 |
|  | 11-12 years | 48 (30.6) | 1.219 (0.594-2.504) | .59 | 1.346 (0.603-3.004) | .47 | 55 (35.0) |  |  | 2.010 (1.049-3.853) | *.04* |
|  | >12 years | 246 (29.6) | 1.164 (0.607-2.234) | .65 | 1.258 (0.605-2.617) | .54 | 309 (37.2) |  |  | 2.144 (1.191-3.860) | *.01* |
| **Employment** | | | | | | | | | | | |
|  | Employed | 84 (36.4) | reference |  |  |  | 92 (39.8) | reference |  |  |  |
|  | Unemployed | 336 (28.0) | 0.679 (0.505-0.913) | *.01* | 0.664 (0.459-0.960) | *.03* | 438 (36.4) |  |  | 0.792 (0.578-1.084) | .15 |
| **Annual disposable income** | | | | | | | | | | | |
|  | ≤50,000 | 314 (30.2) | reference |  |  |  | 399 (38.4) | reference |  |  |  |
|  | 50,001-100,000 | 61 (28.8) | 0.938 (0.675-1.303) | .68 | 0.991 (0.691-1.421) | .96 | 78 (36.8) |  |  | 0.905 (0.675-1.213) | .50 |
|  | 100,001-150,000 | 24 (30.4) | 0.99 (0.596-1.644) | .97 | 0.937 (0.543-1.617) | .82 | 26 (32.9) |  |  | 0.844 (0.536-1.329) | .46 |
|  | >150,000 | 21 (20.6) | 0.627 (0.384-1.025) | *.04* | 0.494 (0.286-0.854) | *.01* | 27 (26.5) |  |  | 0.661 (0.439-0.995) | *.05* |
| **Perceived risk of being infected with HIV** | | | | | | | | | | | |
|  | Sure | 25 (42.4) | reference |  |  |  | 28 (47.5) | reference |  |  |  |
|  | Very likely | 12 (32.4) | 0.653 (0.276-1.543) | .33 | 0.916 (0.355-2.362) | .86 | 15 (40.5) | 0.948 (0.436-2.058) | .89 | 1.217 (0.535-2.768) | .64 |
|  | Possible | 26 (27.7) | 0.520 (0.262-1.033) | .06 | 0.686 (0.321-1.469) | .33 | 37 (39.4) | 0.827 (0.448-1.528) | .54 | 0.995 (0.518-1.911) | .99 |
|  | Unlikely | 148 (37.0) | 0.799 (0.459-1.391) | .43 | 0.842 (0.443-1.599) | .60 | 185 (46.3) | 1.154 (0.688-1.936) | .59 | 1.098 (0.623-1.934) | .75 |
|  | Impossible | 209 (24.8) | 0.448 (0.261-0.769) | *.004* | 0.523 (0.281-0.975) | *.04* | 265 (31.4) | 0.527 (0.320-0.867) | *.01* | 0.564 (0.327-0.972) | *.04* |
| **Sexual orientation** | | | | | | | | | | | |
|  | Homosexual | 54 (23.7) | 0.680 (0.488-0.949) | *.02* | 0.925 (0.630-1.356) | .69 | 83 (36.4) | 0.840 (0.642-1.098) | .20 | 1.181 (0.878-1.589) | .27 |
|  | Bisexual | 49 (25.4) | 0.746 (0.526-1.059) | .10 | 0.998 (0.675-1.475) | .99 | 66 (34.2) | 0.624 (0.469-0.832) | *.001* | 0.865 (0.636-1.176) | .35 |
|  | Heterosexual | 317 (31.3) | reference |  |  |  | 381 (37.6) | reference |  |  |  |
| **Do you have multiple sex partners** | | | | | | | | | | | |
|  | Yes | 142 (44.2) | 2.380 (1.837-3.083) | *<.001* | 2.041 (1.442-2.890) | *<.001* | 157 (48.9) | 1.969 (1.553-2.496) | *<.001* | 1.651 (1.208-2.258) | *.002* |
|  | No | 278 (25.0) | reference |  |  |  | 373 (33.5) | reference |  |  |  |
| **Having unprotected sex** | | | | | | | | | | | |
|  | Yes | 80 (42.3) | 1.951 (1.425-2.672) | *<.001* | 0.842 (0.551-1.286) | .43 | 86 (45.5) | 1.618 (1.211-2.163) | *.001* | 0.921 (0.628-1.352) | .68 |
|  | No | 340 (27.3) | reference |  |  |  | 444 (35.7) | reference |  |  |  |
| **Home continent** | | | | | | | | | | | |
|  | Africa | 268 (39.5) | reference |  |  |  | 321 (47.3) | reference |  |  |  |
|  | America | 33 (49.3) | 1.488 (0.900-2.462) | .12 | 1.116 (0.643-1.937) | .70 | 29 (43.3) |  |  | 0.758 (0.458-1.253) | .28 |
|  | Europe | 11 (27.5) | 0.582 (0.286-1.184) | .14 | 0.591 (0.276-1.263) | .18 | 10 (25.0) |  |  | 0.429 (0.231-0.795) | *.007* |
|  | Asia | 103 (16.9) | 0.312 (0.240-0.405) | *<.001* | 0.335 (0.251-0.448) | *<.001* | 163 (26.7) |  |  | 0.330 (0.261-0.417) | *<.001* |
|  | Other | 2 (33.3) | 0.767 (0.139-4.216) | .76 | 0.602 (0.102-3.563) | .58 | 2 (33.3) |  |  | 0.272 (0.059-1.252) | .10 |
